# Morphological and Behavioral Changes in the Pathogenesis of a Novel Mouse Model of Communicating Hydrocephalus

**DOI:** 10.1371/journal.pone.0030159

**Published:** 2012-01-24

**Authors:** Allison B. McMullen, Gurlal S. Baidwan, Ken D. McCarthy

**Affiliations:** Department of Pharmacology, University of North Carolina at Chapel Hill, Chapel Hill, North Carolina, United States of America; University of Florida, United States of America

## Abstract

The Ro1 model of hydrocephalus represents an excellent model for studying the pathogenesis of hydrocephalus due to its complete penetrance and inducibility, enabling the investigation of the earliest cellular and histological changes in hydrocephalus prior to overt pathology. Hematoxylin and eosin staining, immunofluorescence and electron microscopy were used to characterize the histopathological events of hydrocephalus in this model. Additionally, a broad battery of behavioral tests was used to investigate behavioral changes in the Ro1 model of hydrocephalus. The earliest histological changes observed in this model were ventriculomegaly and disorganization of the ependymal lining of the aqueduct of Sylvius, which occurred concomitantly. Ventriculomegaly led to thinning of the ependyma, which was associated with periventricular edema and areas of the ventricular wall void of cilia and microvilli. Ependymal denudation was subsequent to severe ventriculomegaly, suggesting that it is an effect, rather than a cause, of hydrocephalus in the Ro1 model. Additionally, there was no closure of the aqueduct of Sylvius or any blockages within the ventricular system, even with severe ventriculomegaly, suggesting that the Ro1 model represents a model of communicating hydrocephalus. Interestingly, even with severe ventriculomegaly, there were no behavioral changes, suggesting that the brain is able to compensate for the structural changes that occur in the pathogenesis of hydrocephalus if the disorder progresses at a sufficiently slow rate.

## Introduction

Hydrocephalus is a highly prevalent disorder that accounts for greater than 0.6% of the hospital admissions in the United States, and health care costs related to hydrocephalus cost the U.S. $1.4–2.0 billion annually [1]. Despite its prevalence, the primary treatment for hydrocephalus—the placement of shunts to drain the excess cerebrospinal fluid (CSF)—has not been significantly improved in over fifty years. Moreover, these shunts have a high failure rate—more than two-thirds of shunts fail within a ten-year period [2], [3]. As a result, it is clear that better treatments, such as drug-based therapeutics, are necessary to treat patients with hydrocephalus. However, to create novel treatments for hydrocephalus and to improve the early diagnosis of this disorder, a better understanding of the pathogenesis and underlying causes of hydrocephalus are necessary.

Although the prevalence of congenital hydrocephalus is approximately 0.4–0.8 in 1000 live births, our understanding of the genetic causes of hydrocephalus is extremely limited [4]–[7]. Therefore, the study of genetic animal models of hydrocephalus will likely improve our understanding of the underlying causes and pathogenesis of congenital hydrocephalus and may lead to drug-based therapeutics to treat this disorder. Many genetic animal models for studying hydrocephalus have been developed, including both models of communicating and non-communicating hydrocephalus. Non-communicating hydrocephalus results from a blockage within the ventricular system. In contrast, in communicating hydrocephalus, the passages of the ventricular system remain patent.

Among the first the genetic animal models developed was the hydrocephalus Texas (H-Tx) rat, which was created through spontaneous genetic mutations and has a penetrance of approximately 40% [8]. Previous studies have demonstrated stenosis of the aqueduct of Sylvius in H-Tx rats, suggesting that this is a model of non-communicating hydrocephalus [9], [10]. In contrast, Hy3 mice show defective CSF reabsorption, resulting in communicating hydrocephalus with perinatal onset [11], [12]. In both of these models, loss of the ependymal cells that line the lateral ventricles appears to be the result of ventricular enlargement; as the ventricles enlarge, the ependyma stretches and denudes [13], [14]. The hydrocephalus with hop gait (hyh) mouse model has also been widely described [15]–[20]. This non-communicating model of hydrocephalus displays aqueductal stenosis, and the development of hydrocephalus in this model appears to be dependent on ependymal denudation [16], [17], [19], [20]. However, all of these models either have incomplete penetrance or concomitant pathologies unrelated to hydrocephalus. As a result, it has been impossible to specifically investigate the pathogenesis of hydrocephalus using a genetic animal model.

To address these concerns, our lab has developed a novel model of hydrocephalus based on the Ro1 RASSL (receptor activated solely by a synthetic ligand) [21]. The Ro1 receptor is a transgenic Gi-coupled G protein-coupled receptor (GPCR), which was developed by mutating the kappa opioid receptor (KOR) [22]. The Ro1 receptor no longer responds to endogenous ligands. We used a tetracycline-inducible system to drive Ro1 expression selectively to astrocytes by crossing two single-transgenic lines of mice: one line expresses the tetracycline transactivator (tTA) driven by the glial fibrillary acidic protein (GFAP) promoter, and the other line uses the tetO minimal promoter to drive Ro1 expression. Upon the removal of doxycycline (dox) from the drinking water of double-transgenic KOR knockout mice, tTA binds to the tetO promoter, driving Ro1 expression. Interestingly, we found that when mice are taken off dox, 100% of double-transgenic mice develop hydrocephalus [21]. Additionally, we have demonstrated using immunohistochemistry that the Ro1 receptor co-localizes with astrocyte-specific, but not neuron-specific, markers [21], and data from our laboratory have demonstrated that the Ro1 receptor is also expressed in ependymal cells [23]. Thus, basal levels of Ro1 receptor activity in astrocytes and ependymal cells are enough to cause hydrocephalus in double-transgenic Ro1 mice. The high basal activity levels and high levels of Ro1 expression are likely sufficient to stimulate aberrant GPCR signaling in astrocytes, leading to hydrocephalus. Interestingly, selective expression of the Ro1 receptor in the mouse heart causes lethal cardiomyopathy [24], and its expression in osteoblasts causes trabecular osteopenia [25], both of which are solely due to the basal levels of activity of the Ro1 receptor.

To our knowledge, the Ro1 model is the first model of hydrocephalus to implicate aberrant GPCR signaling in the pathogenesis of this disorder. Additionally, by targeting the Ro1 receptor selectively to GFAP-positive cells (astrocytes and ependymal cells), we have developed a novel model of communicating hydrocephalus in which a single signaling system in a specific cell type leads to hydrocephalus. Thus, due to its inducibility, 100% penetrance and cell-specific, signaling-mediated pathogenesis, the Ro1 model is unique in that it enables the investigation of the early histological, behavioral and signaling events that cause hydrocephalus, without complications from secondary pathologies. The Ro1 model therefore enables researchers to investigate the etiology of hydrocephalus, rather than being restricted to studying pathological changes subsequent to overt hydrocephalus. Findings from the Ro1 model may therefore lead to the development of drug-based therapeutics to treat this disorder. In this study, we used a combination of histology, immunohistochemistry, electron microscopy and behavior to elucidate the histological and behavioral changes that are occur as hydrocephalus progresses.

## Materials and Methods

### Animals

This study was conducted in strict accordance with the recommendations in the Guide for the Care and Use of Laboratory Animals at the National Institutes of Health. All of the experiments were approved by the Institutional Animal Care and Use Committee at the University of North Carolina at Chapel Hill under protocol number 09-096. Animals were maintained in climate-controlled housing with a 12 h light/dark cycle and were given food and water *ad libitum*. Selected breeding pairs and weaned mice were given 50 µg/mL doxycycline (dox; Sigma-Aldrich, St. Louis, MO) in their drinking water, which was changed twice a week. Dox was provided in black water bottles to protect the dox from light. Breeding pairs maintained off dox were never exposed to dox.

### Transgenic mice

GFAP-tTA::tetO-Ro1 mice on a KOR knockout background were generated as described previously [21]. All of the mice were backcrossed onto the C57Bl/6J background (10 generations).

### Real-time PCR

Two 1 mm thick coronal brain sections at the level of the lateral ventricles from six single-transgenic control and six double-transgenic Ro1 mice at 9 days off dox, were sliced on an acrylic matrix and placed in RNA later (Ambion, Austin, TX) at −20°C. RNA was isolated using the RNeasy Lipid Tissue Mini Kit (Qiagen, Valencia, CA), and the amount of RNA per sample was quantified using a NanoDrop spectrophotometer (Thermo Fisher Scientific, Waltham, MA). RNA from each mouse was sent for an RNA quality check at the Nucleic Acids Core Facility at the University of North Carolina at Chapel Hill. cDNA was then prepared using Superscript II Reverse Transcriptase with random primers and RNaseIn to block RNase activity (Ambion, Austin, TX). Real-time PCR was conducted using hKOR2 and GAPDH primers and a TaqMan® Universal PCR Master Mix (Applied Biosystems, Foster City, CA). Fold enrichment was determined using the 2^−ΔCT^ method, and the data are presented as the enrichment of Ro1 expression relative to the lowest expresser.

### Hematoxylin and eosin staining

Single-transgenic controls and double-transgenic Ro1 mice were taken off dox at P30 and transcardially perfused with 4% paraformaldehyde at 9 (controls, n = 4; Ro1, n = 4), 12 (controls, n = 7; Ro1, n = 7), 18 (controls, n = 6; Ro1, n = 6), 24 (controls, n = 6 controls; Ro1, n = 6), 30 (controls, n = 5; Ro1, n = 5) and 48 days (controls, n = 21; Ro1, n = 17) following dox removal. This wide range of time points was chosen to analyze changes in hydrocephalus beginning shortly after Ro1 receptor expression through overt hydrocephalus and moribundity. To study the effect of Ro1 expression throughout gestation and development, dams were maintained off dox, and weaned mice were perfused at P27 (controls, n = 6; Ro1, n = 6). Following perfusion, the brains were removed and post-fixed overnight at room temperature in 10% formalin with gentle agitation. The brains were then rinsed in distilled water and stored in 70% ethanol. The brains were taken to the University of North Carolina at Chapel Hill histology core facility for paraffin embedding. Serial coronal sections were sliced 5 µm thick on a sliding microtome (Leica, Wetzlar, Germany) and stained with hematoxylin and eosin. Stained brain sections were imaged with a light microscope (Zeiss, Oberkochem, Germany), and images were captured using a Dage Excel XL16 camera (Dage-MTI, Michigan City, IN).

### Measurements

Images from hematoxylin and eosin stained coronal brain sections from both single-transgenic controls and double-transgenic Ro1 mice at 0.38 mm anterior to bregma were analyzed with MetaMorph image analysis software (Molecular Devices, Sunnyvale, CA). The maximum width of the lateral ventricles was measured and divided by the maximum brain width to obtain the lateral ventricle to brain ratio (LV∶brain ratio). For measurements of ependymal disorganization at the aqueduct, images of the aqueduct of Sylvius were captured from both control and Ro1 mice at 18, 24, 30 and 48 days off dox immediately posterior to the dorsal third ventricle (approximately 2.92 mm posterior to bregma). Ependymal disorganization was defined as the displacement or loss of the ependymal cells lining the aqueduct and their cilia and was scored by three observers blind to genotype based on the continuity of the ependyma and the degree of cilia coverage. A four-point scoring system was used to classify the aqueductal ependyma as (3) normal, representing a continuous ependymal lining and a dense, continuous carpet of cilia, (2) mild, suggesting minimal ependymal and cilia loss, (1) moderate, representing considerable ependymal and cilia loss, or (0) severe, which represented almost complete loss of ependymal cells and their associated cilia.

### Immunohistochemistry

Single-transgenic controls and double-transgenic Ro1 mice were taken off dox at P30 and transcardially perfused with 4% paraformaldehyde at 9 (controls, n = 4; Ro1, n = 4), 12 (controls, n = 3; Ro1, n = 3) and 24 days (coronal: controls, n = 5; Ro1, n = 5; sagittal: controls, n = 5; Ro1, n = 5) after the removal of dox. These time points were chosen to observe changes in the subventricular zone (SVZ) early in the pathogenesis of hydrocephalus to determine whether changes in this region play a causative role in the development of hydrocephalus in the Ro1 model. The brains were post-fixed for 4 h in 4% paraformaldehyde at 4°C and subsequently transferred to a 30% sucrose solution in PBS (pH 7.4) at 4°C for cryoprotection. The brains were then embedded and frozen in O.C.T. (Optimal Cutting Temperature; Tissue-Tek, Sakura, Japan) in an ethanol/dry ice bath and stored at −80°C. Ten micrometer thick coronal and sagittal slices were cut on a cryostat (Leica, Wetzlar, Germany) and melted onto Superfrost Plus slides (Thermo Fisher Scientific, Waltham, MA). The slices were washed three times with phosphate buffered saline (PBS) and incubated in blocking solution (10% normal goat serum and 0.3% TritonX-100 in PBS). The slices were then incubated in primary antibody in blocking solution overnight at 4°C. The primary antibodies used were doublecortin (DCX, 1∶500; Cell Signaling, Beverly, MA) to stain for migrating neuroblasts, glial fibrillary acidic protein (GFAP, 1∶500; Sigma-Aldrich, St. Louis, MO) to stain for astrocytes and S100 (1∶500; DAKO, Carpenteria, CA) to stain for ependymal cells. The slices were then washed six times for 10 min in PBS and incubated in goat anti-rabbit 594 or goat anti-mouse 488 secondary antibodies (Alexa Fluor, 1∶400; Invitrogen, Carlsbad, CA) in blocking solution for 2 h at room temperature. The slices were washed six times for 10 min in PBS and mounted in Vectashield with DAPI (Vector Laboratories, Burlingame, CA), coverslipped and sealed with nail polish. The sections were imaged with a fluorescent microscope (Zeiss, Oberkochem, Germany).

### Electron microscopy

Single-transgenic controls and double-transgenic Ro1 mice were taken off dox at P30 and transcardially perfused at 18 (controls, n = 8; Ro1, n = 8) and 30 days (controls, n = 4; Ro1, n = 4) off dox with Ringer's solution (1.36 M NaCl, 0.127 M Na_2_HPO_4_, 0.503 M KCl, 0.098 M MgCl_2_, 0.595 M NaHCO_3_, 0.204 M CaCl_2_ and 2.2 mM dextrose, with 0.004% xylocaine to anesthetize smooth muscle and 5 U/mL heparin to prevent blood clotting) followed by 2% paraformaldehyde and 2.5% glutaraldehyde in 0.1 M phosphate buffer for 10 min at a rate of 3 mL/min. These two time points were chosen to investigate the ultrastructural changes immediately prior to ventriculomegaly (18 days off dox) and those present with mild to moderate ventriculomegaly (30 days off dox). The brains were post-fixed in the same fixative for 1 h on ice and sliced 50 µm thick on a Vibratome (Leica, Wetzlar, Germany) in ice-cold fixative. The tissue slices were then post-fixed overnight in the same fixative at 4°C. The sections were subsequently post-fixed in 0.5% osmium tetraoxide in 0.1 M phosphate buffer for 45 min and stained with 1% uranyl acetate in maleate buffer for 45 min. After dehydrating in an ascending ethanol series, the sections were embedded in Epon/Spurr resin (Electron Microscopy Services, Hatfield, PA) and mounted between sheets of Aclar within glass slides. After embedding, the sections were cut 60 nm thick on an Ultracut (Leica, Wetzlar, Germany), mounted onto T200 copper grids (Electron Microscopy Services, Hatfield, PA) and post-stained with 2% uranyl acetate and Sato's lead.

### Behavior

To investigate progressive changes in weight and general motor ability, mice (controls, n = 11; Ro1, n = 10) were taken off dox at P30, and weight, grip strength (wire hang), motor coordination (rotarod), exploratory behavior (open field) and startle responses and sensorimotor gating (acoustic startle) were measured immediately following the removal of dox and every week thereafter for a total of seven weeks to evaluate the behavioral changes that may occur as hydrocephalus develops. For the wire hang test, the mice were placed, one at a time, on a wire grid and inverted approximately 20 cm above a soft sponge. The latency to fall off of the grid was recorded; the maximum cut-off time was 60 s. For the rotarod test, the mice were placed on an accelerating cylinder (rotarod), and the latency to fall off of the rotarod was recorded; the maximum cut-off time was 300 s. The mice were first given a series of three trials to evaluate motor learning; on each subsequent testing day, the mice were given two trials on the accelerating rotarod to measure motor coordination. For the open field test, the mice were placed in a large plexiglas enclosure with a photobeam array (Accuscan Instruments, Columbus, OH) and allowed to freely explore for 1 h. Total distance traveled (cm), horizontal activity, stereotypy counts and rearing activity were measured. For the acoustic startle test, the SR-LAB startle response system was used (San Diego Instruments, San Diego, CA). Briefly, the mice were placed in a Pplexiglas cylinder with white noise at a level of 70 dB. The mice were given a loud startle (120 dB) or a prepulse (74, 78, 82, 86 or 90 dB) followed by the loud startle (120 dB), and the force displaced by a whole-body startle response was measured by an accelerometer. Responses to the startle alone or the prepulse followed by the startle were randomized; the response to each stimulus was tested six times with 15 s between stimuli.

A second cohort of mice (control, n = 10; Ro1, n = 11) was used to test learning and memory in the Morris water maze. The Morris water maze consists of a circular pool (diameter = 122 cm) partially filled with water (45 cm deep, 24–26°C) containing non-toxic white paint. The pool was visually divided into four quadrants. Visual cues were placed in the room surrounding the pool. Visual learning was tested at 10–12 days after removal of dox by placing the mice in the pool and measuring their latency to swim to a visible platform. Learning acquisition of the location of a hidden platform (diameter = 12 cm) was then tested until the average latency of the mice was less than 15 s. This test was conducted from 15–19 days off dox by placing the escape platform below the surface of the water and measuring their latency to learn the location of the hidden platform based on the visual cues in the surrounding room. A probe trial was then conducted at 19 days off dox by measuring the time spent in each of the quadrants; increased time spent swimming in the quadrant where the platform had been previously located (quadrant 1) was considered indicative of learning. The platform was then moved to the opposite quadrant (quadrant 3), and the time required to learn the new location of the platform was measured. The mice were trained in the reversal learning paradigm until the latency to find the hidden platform was, on average, less than 15 s. Reversal learning occurred from 22–32 days off dox, and the probe trial for reversal learning was conducted at 32 days off dox. The mice were then tested for the ability to remember the location of the hidden platform (quadrant 3) by recording their latency to find the platform at 46–47 days off dox (retention). This test was scheduled such that changes in learning and memory (hidden and reversal tests) were evaluated prior to ventriculomegaly through the development of ventriculomegaly. The retention test was timed to evaluate changes in memory with severe ventriculomegaly.

A third group of mice (control, n = 17; Ro1, n = 13) was used to test the development of anxiety and depression in early hydrocephalus. At 24 days off dox, at which point minimal to mild ventriculomegaly is present in Ro1 mice, the mice were tested on the elevated plus maze, which has two open arms and two closed arms (20 cm in height). The maze is elevated 50 cm from the floor and the arms are 30 cm long. The mice were placed in the center section (8 cm×8 cm) and allowed to freely explore the maze. The time spent in the open and closed arms and the number of entries into each of the arms was recorded. The mice were subsequently tested in the forced swim test at 25 days off dox. In this test, the mice were placed in a cylinder (28 cm high; 20.2 cm in diameter) filled with water (14.5 deep; 23°C), and their time spent immobile in a 6 min period was recorded. The time spent in the center of the open field apparatus (AccuScan Instruments, Columbus, OH) in 1 h was used as a measure of anxiety in mice 31 days off dox. Lastly, passive avoidance was tested in mice 32–33 days off dox. The passive avoidance test consisted of two trials. On the first day of testing, the mice were placed in the apparatus, which consists of a light chamber and a dark chamber, separated by a door. After a 30 s acclimation period, the door is opened, and when the mice enter the dark chamber, they receive a mild footshock (0.5 mA, 2 s). On the second day of testing, the latency to enter the dark chamber was measured; a 300 s cut-off was used. Based on histological findings, these final two tests were timed to investigate changes in Ro1 mice off dox with mild to moderate ventriculomegaly.

### Statistics

For comparisons between control and Ro1 mice at a single time point, Student's t-test was used. To analyze changes in the behavioral tests over time as well as changes in the acoustic startle test, repeated-measures ANOVAs were used. For the rotarod and wire hang tests, which were not normally distributed, the Mann Whitley test with the Bonferroni correction was used to compare changes at each time point. An F-test was used to calculate a p-value for the linear regression analyses. P-values<0.05 were considered to be statistically significant. StatView statistical software (version 5.0) was used for all of the statistical tests (SAS, Cary, NC).

## Results

### Ventriculomegaly and disorganization of the aqueduct of Sylvius are the first signs of pathology in the Ro1 model

**Figure 1:**
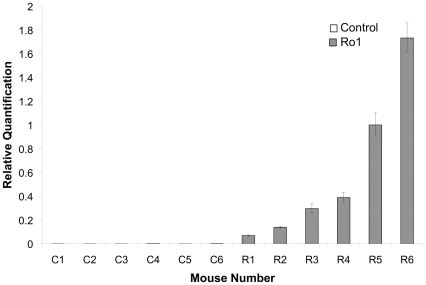
Variable expression of the Ro1 receptor in Ro1 mice at 9 days off doxycycline. This graph shows the relative quantification of Ro1 receptor expression in Ro1 mice and age-matched littermate controls (n = 6). Each sample was run in triplicate, and the error bars show the variability in detected Ro1 expression for each mouse, with the positive error bar representing the maximum value and the negative error bar representing the minimum value. Although the Ro1 receptor was clearly expressed by Ro1 mice by 9 days after removal of dox at P30, there was considerable variability in the levels of expression among Ro1 mice.

**Figure 2:**
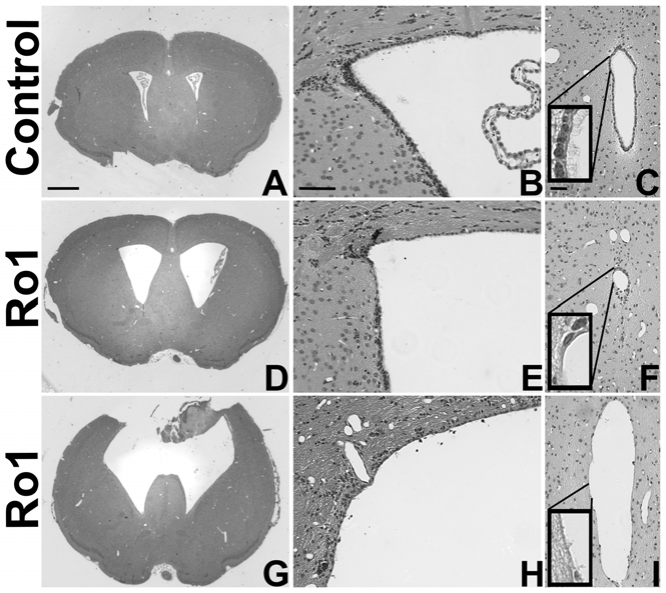
Histopathological changes in the pathogenesis of hydrocephalus in the Ro1 model. Double-transgenic Ro1 mice and age-matched littermate controls were taken off dox at P30 and analyzed at 9 (n = 4), 12 (n = 7), 18 (n = 6), 24 (n = 6), 30 (n = 5) and 48 days (controls, n = 21; Ro1, n = 17) off dox to investigate histopathological changes in the pathogenesis of hydrocephalus in the Ro1 model. Images of coronal brain slices (A, D and G; scale bar = 1.0 mm), the dorsolateral wall of the left lateral ventricle (B, E and H; scale bar = 75 µm) and the aqueduct of Sylvius (C, F and I; scale bar = 50 µm) are shown from a control mouse (A–C; 24 days off dox) and two Ro1 mice (D–F and G–I, 24 and 48 days off dox, respectively). The Ro1 mice showed progressive enlargement of the lateral ventricles (D and G), thinning (E and H) of the ependymal layer of the lateral ventricles and disorganization of the ependymal lining of the aqueduct of Sylvius (F and I).

**Figure 3:**
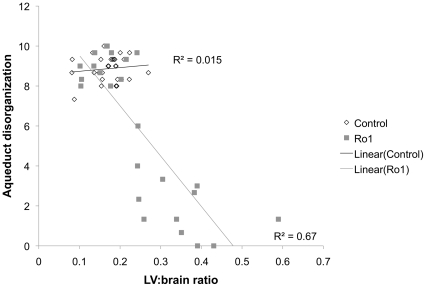
Lateral ventricle size correlates with the degree of ependymal disorganization at the aqueduct of Sylvius. The lateral ventricle size of Ro1 mice is significantly correlated to disorganization of the ependymal cells at the aqueduct of Sylvius (r^2^ = 0.78; p = 6.15 * 10^−9^). A four-point scoring system was used to classify the aqueductal ependyma as (3) normal, representing a continuous ependymal lining and a dense, continuous carpet of cilia, (2) mild, suggesting minimal ependymal and cilia loss, (1) moderate, representing considerable ependymal and cilia loss, or (0) severe, which represented almost complete loss of ependymal cells and their associated cilia. As a reference, panel F of Figure 2 was scored as moderately disorganized (1), whereas panel I of Figure 2 was scored as severely disorganized (0). As lateral ventricle size increased (an increase in the lateral ventricle to brain ratio), the aqueduct became increasingly disorganized.

Although there was clear, but variable, Ro1 receptor expression in the double-transgenic mice by 9 days off dox (Figure 1; n = 6), there were no detectable morphological changes in these mice at 9, 12 or 18 days off dox. Ventriculomegaly was first observed in the Ro1 mice at 24 days off dox (Figure 2). However, likely due to the variability observed in Ro1 receptor expression among the double-transgenic mice, there was considerable variability in ventricle size among the Ro1 mice at this time point (average LV∶brain ratio = 0.22; range = 0.14–0.39). Ventriculomegaly was more pronounced in the Ro1 mice at 30 and 48 days off dox (Figure 2), with an average LV∶brain ratio of 0.25 at 30 days (range = 0.15–0.43) and 0.38 at 48 days (range = 0.18–0.64). Concomitant with the increase in ventricle size, disorganization of the ependymal cells and their cilia was observed at the aqueduct of Sylvius. The degree of ependymal disorganization at the aqueduct was highly correlated with the severity of ventriculomegaly, as measured by the lateral ventricle to brain ratio (r^2^ = 0.78; p = 6.15 * 10^−9^; Figures 2 and 3). However, complete closure of the aqueduct of Sylvius was never observed.

### Ependymal denudation of the lateral ventricles is secondary to ventriculomegaly

Ependymal denudation of the lateral ventricles often accompanies enlargement of the lateral ventricles in humans and other animal models. Interestingly, when taken off dox at P30, the brains from the Ro1 mice rarely showed denudation; denudation was only present at the dorsal surface of the lateral ventricle with severe ventriculomegaly. However, the ependymal lining became thinner across the dorsal, medial and lateral walls of the lateral ventricles with increasing lateral ventricle size (Figure 2). The thinning of the ependymal lining observed at the lateral ventricles differed from the considerable ependymal cell loss and displacement observed at the aqueduct of Sylvius.

### Despite ventriculomegaly, there is no change in subventricular zone organization in early hydrocephalus

**Figure 4:**
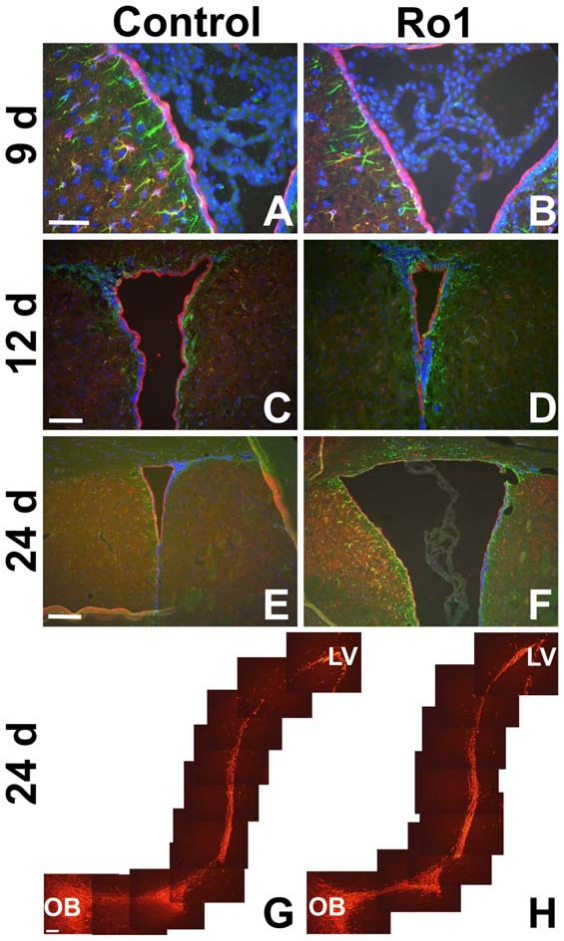
Lack of cellular changes in subventricular zone organization in early hydrocephalus in Ro1 mice. (A) and (B) show GFAP (green) and S100 (red) expression at 9 days off dox in control and Ro1 mice, respectively (n = 4; scale bar = 50 µm). (C) and (D) show GFAP (green) and S100 (red) expression at 12 days off dox in control and Ro1 mice, respectively (n = 3; scale bar = 100 µm). (E) and (F) show GFAP (green) and S100 (red) expression at 24 days off dox of control and Ro1 mice, respectively (n = 5; scale bar = 200 µm). These images were taken from the right lateral ventricle in coronal slices. There were no qualitative changes in GFAP or S100 expression at any time point analyzed. (G) and (H) show DCX (red) expression from the lateral ventricle to the olfactory bulb in sagittal sections from control and Ro1 mice, respectively, at 24 days off dox. There were no qualitative changes in DCX expression or migration at 24 days off dox (n = 5; scale bar = 100 µm).

Because Ro1 expression is being driven to GFAP-positive cells and GFAP is expressed in both subventricular zone (SVZ) astrocytes and at low levels in ependymal cells [23], [26], we hypothesized that Ro1 expression might be affecting SVZ organization. Furthermore, SVZ astrocytes extend processes that both integrate into the ependymal layer and contact blood vessels [27], positioning them to play a potential role in ventriculomegaly and ependymal denudation in hydrocephalus. These SVZ astrocytes are also the progenitor cells of the SVZ, giving rise to neuroblasts that migrate to the olfactory bulb where they form olfactory interneurons [28]–[30]. Moreover, astrocytes direct the migration of the SVZ-born neuroblasts [31]–[33]. To determine whether Ro1 expression in these cells produces changes in SVZ organization and neuroblast migration early in the pathogenesis of hydrocephalus, coronal and sagittal slices were stained for cellular markers of the SVZ. Although there was lateral ventricle enlargement and thinning of the ependymal layer in mice at 24 days off dox, there were no qualitative changes in the staining of astrocytes or ependymal cells between the Ro1 mice and age-matched, single-transgenic controls at 9 (n = 4), 12 (n = 3) or 24 days (n = 5) off dox (Figure 4A–F). Additionally, sagittal slices stained with DCX showed no qualitative changes in the number or migration of SVZ-born neuroblasts between Ro1 mice and age-matched, single-transgenic controls at 12 (data not shown; n = 3) or 24 days (n = 5) off dox (Figure 4G–H).

### Ventriculomegaly leads to thinning of the ependymal layer, which is associated with edema and patches of the ventricular wall barren of cilia and microvilli

**Figure 5:**
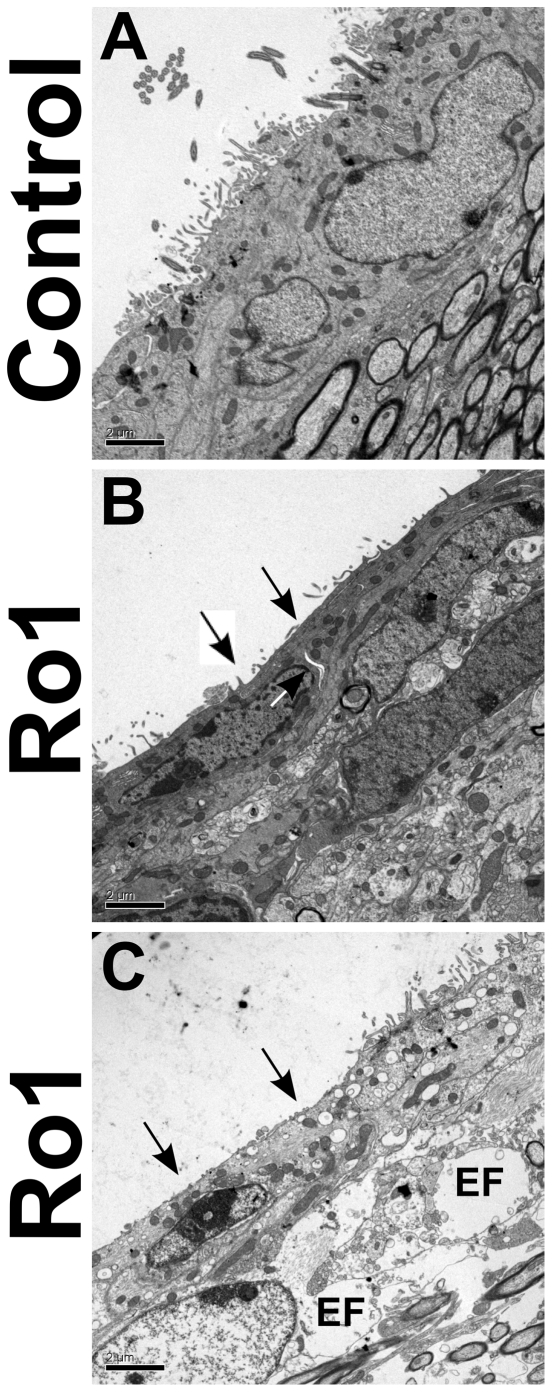
Ultrastructural changes in the periventricular region in Ro1 mice. Ventriculomegaly leads to thinning of the ependymal layer, which was associated with edema and patches of the ventricular wall barren of cilia and microvilli. An age-matched littermate control (A) and two Ro1 (B and C) mice at 30 days off dox are shown (controls, n = 4; Ro1, n = 4). Ro1 mice showed considerable thinning of the ependymal layer and a reduction in the number of microvilli lining the ventricles (black arrows). In the Ro1 mouse with an enlarged head and moderate ventriculomegaly (B), spaces appear to be developing between cells (white arrows). In the Ro1 mouse with a severely enlarged head and severe ventriculomegaly (C), vacuoles were present in the ependymal cell cytoplasm, and edema was present in the periventricular tissue (EF = edematous fluid). These images were taken from the lateral wall of the lateral ventricle. Scale bar = 2 µm.

Because no gross morphological changes were observed with either H&E staining or immunohistochemistry, transmission electron microscopy (TEM) was performed to investigate the ultrastructure of the ependymal and subependymal layers of the lateral ventricles of Ro1 mice early in the development of hydrocephalus. No changes were observed in the Ro1 mice at 18 days off dox, as compared to age-matched littermate controls, which corroborates the findings from the light microscopy experiments (data not shown; n = 8). However, at 30 days off dox (n = 4), considerable thinning of the ependymal layer was observed, as compared to age-matched littermate controls (Figure 5). Additionally, there were patches of the lateral ventricle walls that were barren of cilia and microvilli (Figure 5). A considerable number of vacuoles was present in the ependymal cells of the hydrocephalic mice (Figure 5). Pockets of edematous fluid were also present in the periventricular area (Figure 5).

### Mice maintained off dox show similar disease progression to mice taken off dox at P30

**Figure 6:**
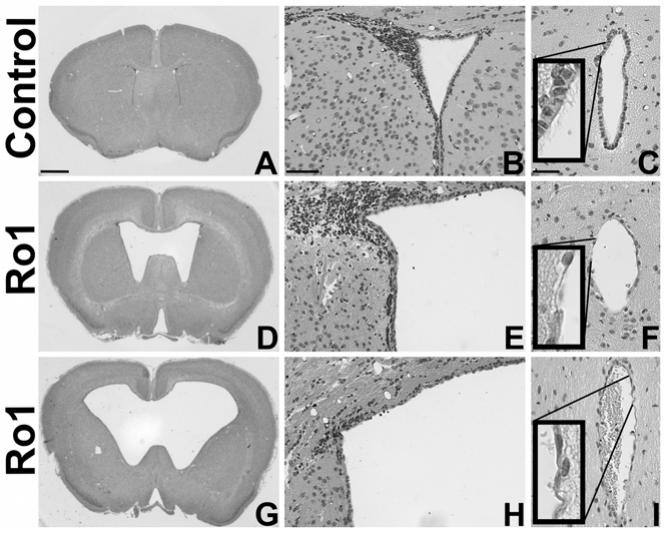
Morphological changes in the progression of hydrocephalus in mice maintained off dox. The progression of hydrocephalus in mice maintained off dox throughout gestation and development mirrors that found in mice taken off dox at P30. Images of coronal brain slices (A, D and G; scale bar = 1.0 mm), the dorsolateral wall of the left lateral ventricle (B, E and H; scale bar = 75 µm) and the aqueduct of Sylvius (C, F and I; scale bar = 50 µm) are shown from a control mouse (A–C) and two Ro1 mice (D–F and G–I) at P27. The Ro1 mice showed variable enlargement of the lateral ventricles (D and G), thinning (E) or loss (H) of the ependymal layer at the lateral ventricles and disorganization of the ependymal lining of the aqueduct (F and I). The average LV∶brain ratio was 0.34, and the range = 0.18–0.64. (n = 4).

To investigate whether Ro1 expression throughout gestation and development alters disease progression in double-transgenic mice, dams were never given dox, and both Ro1 and single-transgenic littermate control pups were maintained off dox until perfusion at P27 (n = 4). Double-transgenic Ro1 mice showed severely enlarged heads at P27 (data not shown). Histological analysis using H&E staining showed severe but variable ventriculomegaly in the Ro1 mice (Figure 6). Ependymal denudation was only present at the roof of the lateral ventricle in Ro1 mice with severely enlarged lateral ventricles. Additionally, despite analysis of serial coronal sections from the third ventricle through the fourth ventricle, complete closure of the aqueduct of Sylvius was never observed even in the most severely hydrocephalic brains studied, although disorganization of the ependymal lining of the aqueduct was present in all of the Ro1 mice with ventriculomegaly (Figure 6).

### Behavioral changes are mostly absent in Ro1 mice despite ventriculomegaly

**Figure 7:**
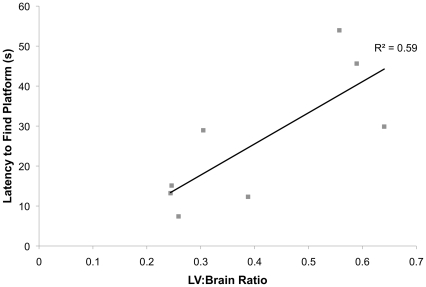
Lateral ventricle size is correlated to the latency to find the hidden platform during retention testing. Testing for memory retention of the location of the hidden platform was performed two weeks following the reversal probe trial. There was a moderate correlation between the lateral ventricle to brain ratio and the latency to find the hidden platform at 46 days off dox (retention, trial 1), suggesting reduced memory in Ro1 mice (r^2^ = 0.59; p = 0.026).

To our surprise, no changes in weight or motor ability were observed in the Ro1 mice over seven weeks of testing (Table 1; control, n = 11; Ro1, n = 10). Subsequent perfusion and histological analysis of the brains of these mice showed moderate to severe ventriculomegaly (LV∶brain ratio >0.3) in 67% of the mice at week 7. However, there were no correlations between lateral ventricle size and behavioral changes for any of the motor tests (data not shown). Weight dropped off precipitously a couple of days prior to death. There were also no significant changes in the performance of the Ro1 mice in the Morris water maze (Table 2; control, n = 11; Ro1, n = 10); however, there was a moderate correlation (r^2^ = 0.59; p = 0.026) between lateral ventricle size and the latency to find the hidden platform in the retention test at 46 days off dox (Figure 7). Additionally, there were no significant changes in any of the behavioral tests measuring anxiety or depression (Table 3; control, n = 17, Ro1, n = 13).

**Table 1:** Changes in weight and general motor ability of Ro1 mice and littermate controls over seven weeks of testing.^a^

	Week 1			Week 4			Week 7			p-value
	Control	Ro1	p-value	Control	Ro1	p-value	Control	Ro1	p-value	(7 weeks)^b^
Weight (g)	12.5±0.5	12.7±0.5	0.77	18.1±0.67	17.7±0.79	0.71	20.3±0.8	19.7±0.8	0.67	0.90
Wire hang	58±2	56±4	0.94	60±0	55±5	0.70	60±0	51±7	0.36	NA^c^
(latency, s)										
Rotarod	219±27	229±23	>0.99	287±7	232±31	0.26	259±18	250±24	0.90	NA^c^
(latency, s)										
Total distance	5790±563	5816±411	0.97	7817±1144	6045±901	0.25	8573±1051	11817±2189	0.16	0.62
(cm)										
Startle response	615.8±58.4	520.9±20.7	0.18	970.5±113.3	899.2±94.3	0.65	862.1±98.6	814.3±102.0	0.75	0.23
(to 120 dB)										

Ro1, n = 10; Control, n = 11.

a Weeks 1, 4 and 7 are shown for simplicity. There were also no significant changes at weeks 2, 3, 5 or 6.

b Student's t-test was used to measure differences between Ro1 and control mice at each week; a repeated measures ANOVA was used to measure changes across all seven weeks of testing. For the wire hang and rotarod tests, which were not normally distributed, the Mann Whitley test was used to compare differences between the Ro1 and control mice at each week.

c Repeated measures ANOVAs across all seven weeks of testing were not calculated for the wire hang and rotarod tests because these data were not normally distributed.

**Table 2:** Performance of Ro1 mice and littermate controls in the Morris water maze.

	Days off dox	Control	Ro1	p-value
Hidden platform acquisition	19	14±2	11±1	0.6
(latency, trial 4, s)				
Hidden probe trial	21	25±3	23±2	0.86
(duration quadrant 1, s)				
Reversal platform acquisition	32	12±2	16±4	0.93
(latency, trial 9, s)				
Reversal probe trial	34	14±1	19±3	0.17
(duration quadrant 3, s)				
Retention	47	18±4	26±6	0.26
(latency, trial 1, s)				

Ro1, n = 10; Control, n = 11.

**Table 3:** Assessment of anxiety and depression in Ro1 mice and littermate controls.

	Days off dox	Control	Ro1	p-value
Elevated plus maze	24	59.5±4.8	61.5±4.9	0.78
(open arm time, s)				
Forced swim test	25	81.6±12.5	74.5±8.7	0.66
(time immobile, s)				
Open field	31	19.6±4.1	25.2±3.8	0.33
(center time, s)				
Passive avoidance	33	68.3±19.1	42.4±9.8	0.45
(latency, s)				

Ro1, n = 13; Control, n = 17.

## Discussion

In this study, we show that selectively expressing the transgenic Ro1 receptor in astrocytes using a tetracycline-inducible system results in a novel and inducible model of hydrocephalus that can be used to study the etiology and the pathogenesis of this disorder. The careful histological analysis of the Ro1 model presented in this study demonstrates that ventriculomegaly and disorganization of the ependyma lining of the aqueduct of Sylvius are the earliest morphological changes observed in the pathogenesis of hydrocephalus in the Ro1 model. Ventricular ependymal denudation and cilia loss, a common feature of hydrocephalus in humans and animal models, appear to result from enlargement of the lateral ventricles. This finding suggests that ventricular ependymal loss and cilia dysfunction are not a cause of hydrocephalus but rather due to the resulting pathology. Furthermore, complete closure of the aqueduct of Sylvius, the narrowest part of the ventricular system, was never observed. Additionally, no obstructions were present in the ventricular system, suggesting that the Ro1 model represents a model of communicating hydrocephalus. It is interesting that although the aqueduct never closed, disorganization of the ependyma of the aqueduct was observed. The mechanism leading to the disorganization of the aqueduct as well as the role of this disorganization in the pathogenesis of hydrocephalus remain unclear. Previous studies have suggested that aqueductal stenosis alone may cause ventriculomegaly, and studies in the hyh mouse model of hydrocephalus have demonstrated that the onset of severe hydrocephalus is associated with aqueductal stenosis caused by the failure of the subcommissural organ to form a normal Reissner's fiber [17], [34]. Although closure of the aqueduct was never observed in this study and is therefore not the cause of hydrocephalus in the Ro1 model, it is possible that the loss of ependymal cells and cilia in the aqueduct in our model may cause hydrocephalus by affecting the bulk flow of CSF through the aqueduct, but further studies are necessary to investigate this possibility.

These histological changes observed in Ro1 model of hydrocephalus appear to mirror human hydrocephalus in many respects. Multiple studies have suggested that ependymal denudation is subsequent to ventriculomegaly and the degree of denudation appears to be dependent on the size of the lateral ventricles and that the rate at which hydrocephalus progresses; more rapid disease progression leads to increased ependymal loss [35]–[38]. We also observed increased ependymal denudation and cilia loss with increasing ventricular size and a correlation between the rate of hydrocephalus development and both denudation and cilia density. Whereas considerable denudation and loss of cilia was found in P27 mice maintained off dox throughout gestation and development, denudation was rarely observed in Ro1 mice taken off dox at P30, even with severe ventriculomegaly. These data suggest that in the Ro1 model, the progression of hydrocephalus occurs more slowly when hydrocephalus is induced by the removal of dox in young adults; this may provide the ependyma with greater time to compensate for the enlargement of the ventricles, thereby reducing the degree of denudation observed.

Interestingly, thinning of the ependymal layer due to ventriculomegaly appears to lead to edema and a loss of microvilli lining the walls of the lateral ventricles. Previous TEM studies in the hy3 mouse model and in a rabbit with kaolin-induced hydrocephalus have also shown stretching of the ependymal layer, intact but widely separated clusters of cilia and microvilli and edematous tissue with enlarged extracellular spaces, which mirrors the results obtained in our study [37], [39]. The variable loss of microvilli and the degree of edema in our model at 30 days off dox is likely due to the highly variable rate of disease progression in these mice; a greater degree of ventriculomegaly was associated with increased edema and loss of microvilli. Studies have suggested that fluid accumulation in the periventricular region, which has been termed periventricular edema, is a result of transependymal flow of CSF due to the loss of tight junctions between the ependymal cells caused by ventriculomegaly [37], [40], [41]. Our TEM data demonstrating intracellular spaces between cells in mice with moderate hydrocephalus and subsequent periventricular edema in mice with severe hydrocephalus support this idea.

Subependymal gliosis is also commonly observed in humans and animals with chronic hydrocephalus. However, subependymal gliosis has been shown to be subsequent to ependymal loss [36], [37]. Additionally, other studies in the H-Tx rat model of congenital hydrocephalus have suggested that ventricular dilation and increased intracranial pressure serve as triggers for gliosis around the periventricular and periaqueductal regions [42], [43]. Thus, it is perhaps unsurprising that we did not observe gliosis early in the pathogenesis of hydrocephalus in Ro1 mice, despite the mild ventriculomegaly present in the Ro1 mice at 24 days off dox. However, a previous study has demonstrated gliosis in the hippocampus and cortex of severely hydrocephalic Ro1 mice [21], suggesting that gliosis occurs in Ro1 mice following severe ventricular dilation. Although changes in the subventricular zone of human fetuses with moderate communicating hydrocephalus have also been observed, these changes were shown to be subsequent to ventricular dilation and ependymal loss [44]. Therefore, it is possible that SVZ disorganization is also present in Ro1 mice, but only in mice with severe hydrocephalus and ependymal denudation.

Surprisingly, there were no behavioral changes observed in the pathogenesis of hydrocephalus in the Ro1 model, even when severe ventriculomegaly was present. Although we were surprised to discover a lack of significant behavioral changes in Ro1 mice even in the presence of ventriculomegaly, our data are perhaps unsurprising considering the slow development of hydrocephalus when Ro1 mice are taken off dox at P30. Del Bigio and colleagues have suggested that a slower rate of disease progression results in less severe morphological and behavioral changes and that there is a threshold of ventricle size beyond which functional changes appear, which is dependent on various factors, such as age of onset and rate of ventricular enlargement [45]. The results of previous studies on behavioral changes in animal models of hydrocephalus have shown conflicting results that appear to be dependent on the severity of hydrocephalus and age of onset. For example, there was no change in rotarod performance in juvenile or adult mice injected with kaolin at 7 or 14 days post-injection [46] or in kaolin-injected neonatal rats at P20, 19 days after kaolin injection [47]. In contrast, when rats were injected with kaolin to induce hydrocephalus at three weeks, there was a significant decrease in rotarod performance at eight months post-injection [45], suggesting that severe hydrocephalus is necessary in the kaolin-injected model before behavioral changes on the rotarod are apparent. Further, changes in the Morris water maze test have only been observed in mice with overt hydrocephalus [48]–[50]. Interestingly, Del Bigio et al. (2002) have demonstrated that there is a significant inverse relationship between ventricle volume and final performance in the water maze [48]. These changes mirror those found in our study: performance in the Morris water maze was not impaired until severe ventriculomegaly was present. Additionally, past behavioral studies in H-Tx rats and rats with kaolin-induced hydrocephalus have suggested that motor activity and body weight rapidly deteriorate immediately prior to death [49], [51]. In our study, we also found hypoactivity, reduced rotarod performance and a loss of body weight in mice immediately prior to death. In humans, gait abnormalities, motor disorders and memory and cognitive deficits are the most common behavioral changes; however, these behavioral deficits are associated with overt hydrocephalus and are likely due to the compression of brain tissue (e.g., the cortex, hippocampus and cerebellum) that is common with severe hydrocephalus [52]–[56].

It is therefore likely that in the Ro1 model, taking the mice off dox to induce astrocyte-specific Ro1 expression and hydrocephalus at P30 results in a slow rate of ventricular enlargement that reduces the likelihood of observing behavioral changes. It is possible that compensation is occurring in the brains of these mice, allowing them to adapt to the gross morphological changes resulting from ventriculomegaly. Considering that we see more rapid disease progression in Ro1 mice maintained off dox throughout gestation and development, it is possible that Ro1 mice taken off dox at a younger age would show a more rapid rate of ventricular enlargement and behavioral changes.

At this point, it remains unclear how the expression of the Ro1 receptor in GFAP-positive cells, namely astrocytes and ependymal cells, leads to hydrocephalus in the Ro1 model. The processes of subependymal astrocytes have been demonstrated to both integrate into the ependymal lining of the ventricular system and contact the vasculature, positioning them to play a central role in the development of hydrocephalus [57]. It is possible that Ro1 expression in astrocytes and ependymal cells affects CSF production or absorption. For example, aquaporin 4 is a water channel that is expressed on astrocytes and ependymal cells at the borders between the brain parenchyma and major fluid compartments, including at astrocyte endfeet, at the glia limitans and at the brain-ventricular CSF barrier [58], [59]. Thus, dysregulation of aquaporin 4 may alter the normal removal of redundant fluid from the parenchyma to the ventricular system and subarachnoid space. Further, knock-out of aquaporin 4 has been shown to exacerbate hydrocephalus in kaolin-induced hydrocephalus, suggesting the importance of aquaporin 4 in water clearance in hydrocephalus [60]. Further studies are necessary to investigate changes in aquaporin 4 expression and other signaling mediators downstream of the Ro1 receptor in the development of hydrocephalus in the Ro1 model.

In sum, the Ro1 model of hydrocephalus represents a novel model of communicating hydrocephalus. This model has many similarities to both human hydrocephalus and experimental models of hydrocephalus. However, the complete penetrance, inducibility and lack of complicating pathologies in this model provide the unique opportunity to investigate the etiology and the pathogenesis of hydrocephalus, which may lead to an understanding of the underlying causes of hydrocephalus. Moreover, because the Ro1 receptor is a GPCR and is restricted to GFAP-positive cells, this model enables the investigation of specific signaling cascades that may cause hydrocephalus, which may lead to the development of novel drug-based therapeutics to treat this disorder. The data presented herein provide insight into the earliest cellular and histological changes in the development and progression of hydrocephalus, providing a well-characterized system in which signaling mechanisms involved in the development of hydrocephalus can be identified. Additionally, the behavioral data suggest that treatments that slow the rate of progression of this disorder may have therapeutic potential by allowing the brain to compensate and increasing the therapeutic window for shunt placement or alternative treatments.
